# LL-37 Attenuates Sepsis-Induced Lung Injury by Alleviating Inflammatory Response and Epithelial Cell Oxidative Injury via ZBP1-Mediated Autophagy

**DOI:** 10.3390/toxins17060306

**Published:** 2025-06-17

**Authors:** Hu Gao, Fajuan Tang, Bin Chen, Xihong Li

**Affiliations:** 1 Department of Emergency Medicine, West China Second University Hospital, Sichuan University, Chengdu 610041, China; drgaohu@163.com (H.G.); kftangfajuan@163.com (F.T.); cb101469@163.com (B.C.); 2 Key Laboratory of Birth Defects and Related Diseases of Women and Children (Sichuan University), Ministry of Education, Sichuan University, Chengdu 610041, China

**Keywords:** sepsis-induced ALI, LL-37, oxidative injury, inflammation, autophagy

## Abstract

**Background:** Sepsis-induced acute lung injury (ALI) is a serious disease constituting a heavy burden on society due to high mortality and morbidity. Inflammation and oxidative stress constitute key pathological mechanisms in ALI caused by sepsis. LL-37 can improve the survival of septic mice. Nevertheless, its function and underlying mechanism in sepsis-evoked ALI is elusive. **Methods:** The human A549 alveolar epithelial cell line was treated with LL-37 or ZBP1 recombinant vector under LPS exposure. Then, the effects on cell oxidative stress injury, inflammatory response, and autophagy were analyzed. RNA-seq analysis was performed to detect the differentially expressed genes (DEGs) between the LPS and LPS/LL-37 groups. Furthermore, the effects of LL-37 on cecal ligation and the puncture (CLP)-constructed ALI model were explored. **Results:** LL-37 attenuated LPS-evoked oxidative injury in human alveolar epithelial cells by increasing cell viability and suppressing ROS, malondialdehyde, and lactate dehydrogenase levels and apoptosis. Moreover, LPS-induced releases of pro-inflammatory IL-18, TNF-α, and IL-1β were suppressed by LL-37. Furthermore, LPS’s impairment of autophagy was reversed by LL-37. RNA-seq analysis substantiated 1350 differentially expressed genes between the LPS and LPS/LL-37 groups. Among them was ZBP1, a significantly down-regulated gene with the largest fold change. Moreover, LL-37 suppressed LPS-increased ZBP1 expression. Importantly, ZBP1 elevation restrained LL-37-induced autophagy in LPS-treated cells and abrogated LL-37-mediated protection against LPS-evoked oxidative injury and inflammation. LL-37 ameliorated abnormal histopathological changes, tissue edema, the lung injury score, oxygenation index (PaO2/FiO2), and glycemia contents in the CLP-constructed ALI model, which were offset through ZBP1 elevation via its activator CBL0137. Additionally, LL-37 suppressed inflammation and oxidative stress in lung tissues, concomitant with autophagy elevation and ZBP1 down-regulation. **Conclusions:** LL-37 may alleviate the progression of sepsis-evoked ALI by attenuating pulmonary epithelial cell oxidative injury and inflammatory response via ZBP1-mediated autophagy activation, indicating a promising approach for the therapy of ALI patients.

## 1. Introduction

Sepsis constitutes life-threatening organ dysfunction and is characterized by a complicated host response to invading pathogenic microorganisms. Due to life-threatening hemodynamic instability, sepsis is a major contributor to mortality in the hospital [1,2]. In China, the incidence of sepsis in intensive care unit (ICU) patients is approximately 25.5% [3]. Sepsis accounts for 13% of hospital costs and over 24 billion clinical burdens in the USA [4]. It is a fact that acute systemic inflammation caused by sepsis will first spread to the respiratory system, where the lungs tend to become the first affected organs, and it is generally believed that approximately half of patients with sepsis develop acute lung injury (ALI) [5]. Notably, ALI and its most severe form, acute respiratory distress syndrome (ARDS), constitute devastating clinical complications arising from sepsis. They are characterized by inflammatory cell infiltration, refractory hypoxemia, pulmonary edema, and serious respiratory failure [5]. Mortality caused by ARDS is over 40%, and it leads to approximately 75,000 deaths annually in the USA [6]. Despite abundant advances, current therapeutic intervention has not been convincingly effective [7].

As a proverbial inflammation-related disease, sepsis-evoked ALI/ARDS chiefly manifests as immune cell infiltration and produces inflammatory mediators, such as TNF-α. Inflammatory cytokines and cells will enter blood circulation and accumulate in the lungs, leading to the release of abundant inflammatory cytokines and oxygen free radicals to further aggravate inflammatory response in the lungs [8,9]. An excessive inflammatory response in ALI will result in pulmonary structural cell injury, especially alveolar epithelial cells [10]. It is known that alveolar epithelial cell damage leads to the dysfunction of the alveolar epithelial barrier, allowing inflammatory cells into the alveolar space from the blood, further exacerbating lung tissue hypoxia and aggravating lung damage in sepsis [5,11]. Oxidative stress is also known as a major pathological mechanism for ALI. During the progression of ALI, the systemic inflammatory response also induces abundant production of ROS to evoke excessive oxidative stress [12]. Oxidative stress accumulation can lead to fluid leakage, alveolar epithelial cell apoptosis, and the penetration of inflammatory cells, exacerbating the progression of ALI [12]. Currently, the targeting of the inflammatory response and oxidative stress represents a promising potential therapeutic strategy in ALI/ADRS [12,13].

It is known that sepsis concerns life-threatening systemic inflammation caused by pathogens, especially bacteria. Antimicrobial peptides constitute the first natural defense barrier blocking invading pathogens in multiple mammalian species, playing important roles in inflammation-related diseases, including sepsis. LL-37, a 37-residue helical peptide, represents the only proverbial human antimicrobial peptide. It is believed that LL-37 is majorly released from the neutrophils and epithelial cells of the testis, gastrointestinal tissue, skin, and respiratory tract [14]. Compared with traditional antibiotics, LL-37 possesses broad antibacterial activity and is not susceptible to drug resistance or immune rejection. Intriguingly, increasing attention has revealed that LL-37 plays important roles in diverse biological processes, including immune regulation, inflammatory response, re-epithelialization, and wound healing [14]. Notably, a previous study confirmed the anti-apoptotic efficacy of LL-37 in LPS-treated human microvascular blood vessel endothelial cells [15]. Also notably, a recent study highlighted that LL-37 attenuates bacterial burden and improved the survival of septic mice [16]. Nevertheless, the roles and underlying mechanism of LL-37 in sepsis-evoked ALI remain undefined.

In the present research, we assessed LL-37’s therapeutic potential using preclinical models. Moreover, the underlying mechanism involved in the above processes was further elucidated.

## 2. Results

### 2.1. LL-37 Alleviates LPS-Induced Oxidative Injury in A549 Cells

**Figure 1:**
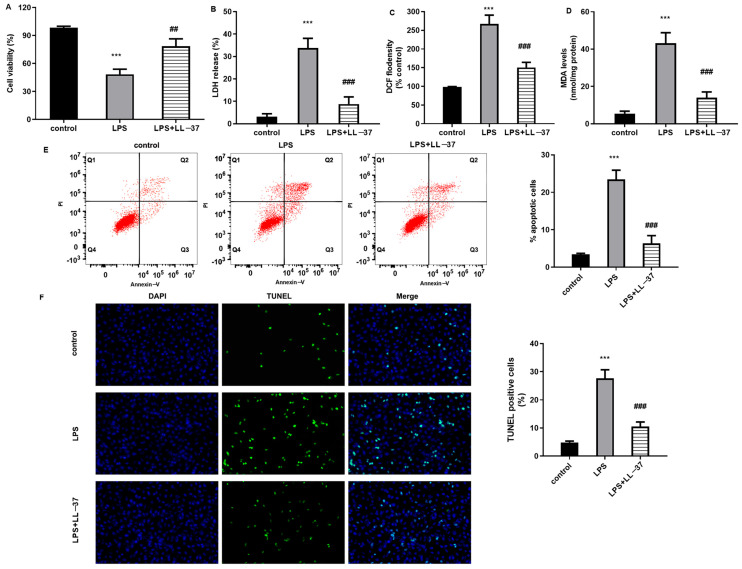
Treatment with LL-37 attenuated oxidative injury in LPS-treated A549 cells. (**A**) A549 cells were treated with LL-37 under LPS exposure (2 μg/mL) or not for 24 h. Then, cell viability was analyzed by a CCK-8 assay. (**B**) The release of LDH from A549 cells was evaluated by a commercial kit. (**C**,**D**) The levels of ROS (**C**) and MDA (**D**) were determined in cells under LPS or LL-37 exposure. (**E**) Cells were stained with annexin V/PI. Then, a flow cytometer was used to analyze cell apoptosis. (**F**) TUNEL staining was applied to determine cell apoptosis. *** *p* < 0.001 vs. control group. ^##^ *p* < 0.01, ^###^ *p* < 0.001 vs. LPS group.

Before the investigation of LL-37 function in sepsis-induced ALI, we first explored the roles of LL-37 in alveolar epithelial cell injury in vitro. As shown in Figure 1A, cell viability was suppressed by LPS, which was reversed by LL-37. Furthermore, LPS exposure elevated the release of LDH (Figure 1B), ROS production (Figure 1C), and MDA levels (Figure 1D). Noticeably, LL-37 attenuated LPS-induced oxidative stress in A549 cells by inhibiting LDH release, ROS, and MDA levels (Figure 1B–D). Additionally, the flow cytometer (Figure 1E) and TUNEL staining (Figure 1F) both confirmed that LPS exposure induced cell apoptosis relative to the control groups. Intriguingly, the LL-37 treatment reversed LPS-evoked cell apoptosis. Thus, these data reveal that LL-37 may antagonize LPS-induced oxidative injury in A549 cells.

### 2.2. LL-37 Inhibits LPS-Evoked Inflammatory Response in A549 Cells

**Figure 2:**
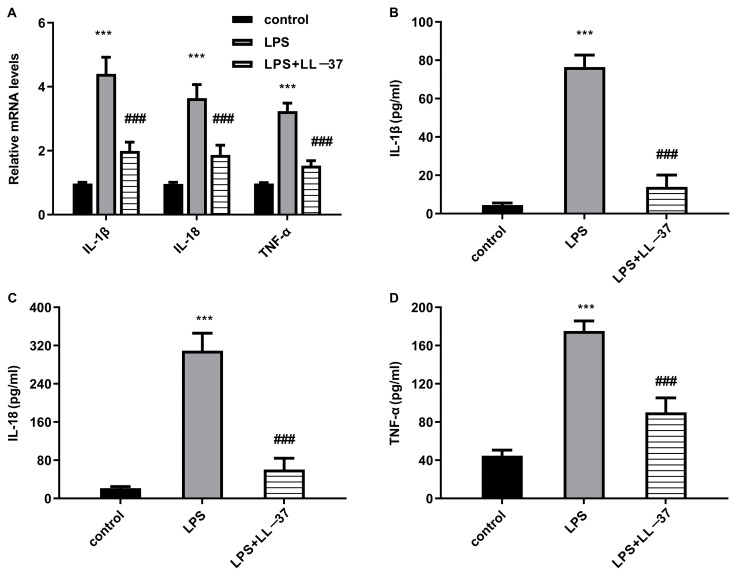
LL-37 inhibited inflammatory response in A549 cells under LPS conditions. (**A**) Cells were exposed to LL-37 under LPS conditions. Then, the transcripts of IL-1β, IL-18 and TNF-α were quantified using a qRT-PCR assay. (**B**–**D**) The levels of IL-1β (**B**), IL-18 (**C**), and TNF-α (**D**) in cell supernatants were determined by an ELISA assay. *** *p* < 0.001 vs. control group. ^###^ *p* < 0.001 vs. LPS group.

As presented in Figure 2A, compared with the control groups, transcripts of pro-inflammatory TNF-α, IL-1β, and IL-18 were increased after LPS exposure. Intriguingly, the treatments with LL-37 restrained the above LPS-induced increases. Additionally, the cells under LPS conditions exhibited higher production of IL-1β (Figure 2B), IL-18 (Figure 2C), and TNF-α (Figure 2D) relative to the control groups. However, LL-37 administration dramatically suppressed the above LPS-induced pro-inflammatory cytokine releases (Figure 2B–D), indicating the anti-inflammatory efficacy of LL-37 in LPS-treated epithelial cells.

### 2.3. LL-37 Enhances Autophagy in LPS-Treated Pulmonary Epithelial Cells

**Figure 3:**
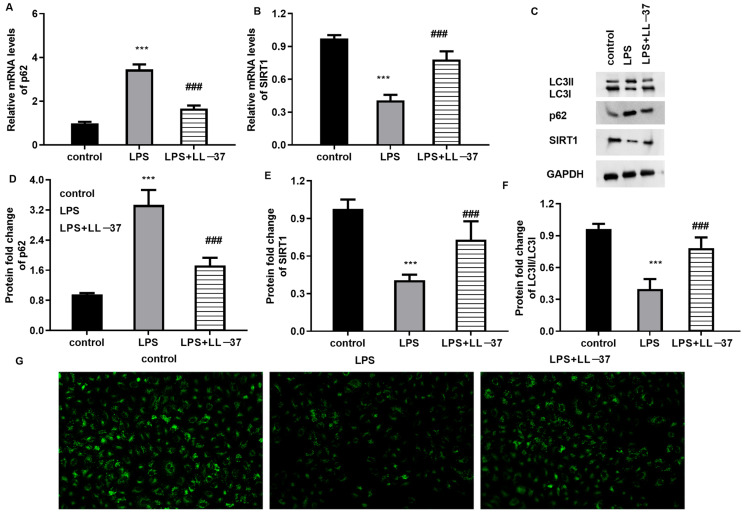
LL-37 enhanced autophagy activation in LPS-stimulated cells. After treatment with LPS or LL-37, the mRNA levels of p62 (**A**) and SIRT1 (**B**) were detected. (**C**–**F**) Then, the protein expression of LC3, p62, and SIRT1 was analyzed by Western blotting (**C**). The corresponding bands were quantified by the Image J software (**D**–**F**). (**G**) Autophagy activation was analyzed by detecting fluorescence levels of LC3II. *** *p* < 0.001 vs. control group. ^###^ *p* < 0.001 vs. LPS group.

Accumulating evidence reveals that autophagic activation exerts an important function in ameliorating the development of sepsis-evoked ALI [17,18]. Thus, we elucidated the correlation between LL-37 and autophagy in LPS-treated cells. As shown in Figure 3A,B, there were higher mRNA levels of autophagy-specific substrate p62 (Figure 3A) and a lower transcription of autophagy-related SIRT1 (Figure 3B) in the LPS-treated cells relative to the control groups. Noticeably, LL-37 offset the above LPS-evoked changes. Moreover, the impaired autophagic activation was validated in the LPS-treated cells as evidenced by decreased LC3II and SIRT 1 expression and increased p62 expression (Figure 3B–F), which were overturned by LL-37. Additionally, fluorescence analysis revealed that LL-37 reversed the suppressive effects of LPS on LC3II fluorescence levels, indicating increased autophagy levels in the LPS-treated cells.

### 2.4. LL-37 Increases Autophagy by Regulating ZBP1 in LPS-Treated Epithelial Cells

**Figure 4:**
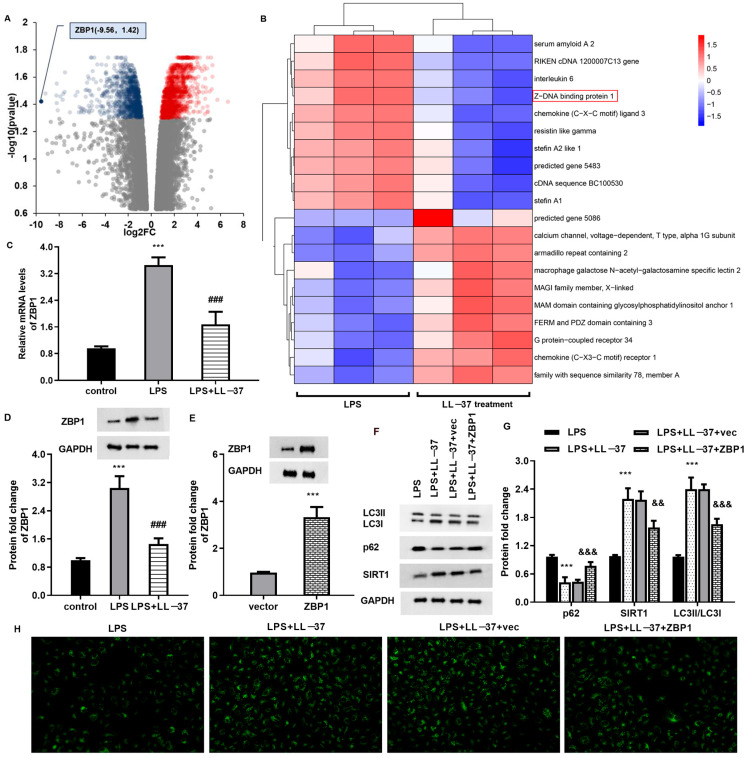
LL-37 regulated autophagy in the LPS-treated cells by inhibiting ZBP1 expression. (**A**) Volcano plot showing DEGs in the LPS and LPS + LL-37 groups. Gray dots indicate no difference, red dots indicate significantly up-regulated genes, and blue dots indicate significantly down-regulated genes. (**B**) Heatmap revealing the top 10 down-regulated and up-regulated genes in these groups. The red box marks ZBP1. (**C**,**D**) The mRNA (**C**) and protein (**D**) levels of ZBP1 were detected in cells under LPS and/or LL-37 conditions. (**E**) The effects of ZBP1 plasmid transfection were evaluated by a Western blotting assay. (**F**–**H**) Cells transfected with ZBP1 vectors were treated with LL-37 under LPS conditions. Then, the protein expression of LC3, p62, and SIRT1 was analyzed (**F**,**G**). LC3 fluorescence levels were then determined (**H**). *** *p* < 0.001 vs. control group. ^###^ *p* < 0.001 vs. LPS group. ^&&^ *p* < 0.05, ^&&&^ *p* < 0.001 vs. LPS and LL-37 group.

To elaborate the underlying mechanism of LL-37 in vitro, we stimulated cells with LPS and/or LL-37 and performed RNA-seq sequencing analysis to quantify the gene expression profiles. Intriguingly, a volcano plot revealed that there were 1350 DEGs between the LPS and LPS + LL-37 groups, including ZBP1 (Figure 4A). Then, the top 10 down-regulated and up-regulated genes were shown as a heat map; among them, ZBP1 was a significantly down-regulated gene with the largest fold change, with a log2FC of −9.56 (Figure 4B). Thus, ZBP1 was chosen for further experiments. Of interest, LPS induced the obvious transcription (Figure 4C) and protein expression (Figure 4D) of ZBP1 in the A549 cells which were abrogated by LL-37. Furthermore, the cells transfected with ZBP1 plasmid confirmed a high expression of ZBP1 (Figure 4E). Importantly, LL-37-mediated increases in autophagy activation were offset after ZBP1 overexpression by decreasing the SIRT1 and LC3II protein levels and increasing p62 expression (Figure 4F,G). Moreover, ZBP1 elevation reversed LL-37-induced increases in LC3II fluorescence levels in the LPS-treated cells, indicating that LL-37 may affect autophagy flux in LPS-treated cells by regulating ZBP1.

### 2.5. ZBP1 Elevation Antagonizes LL-37-Mediated Protection of Epithelial Cells from LPS-Induced Oxidative Injury and Inflammation

**Figure 5:**
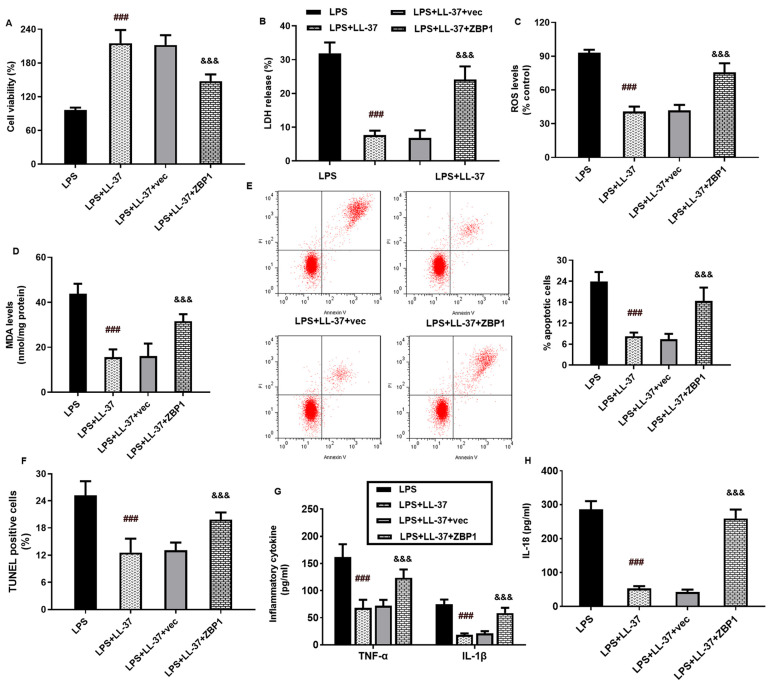
ZBP1 accounted for LL-37-mediated anti-oxidative injury and inflammation in LPS-treated cells. (**A**–**D**) A549 cells were transfected with ZBP1 vectors under LL-37 and LPS conditions. Then, cell viability (**A**), LDH release (**B**), ROS (**C**), and MDA (**D**) levels were measured. (**E**,**F**) Cell apoptosis was determined by annexin V (**E**) and TUNEL (**F**) staining. (**G**,**H**) The production of IL-1β, TNF-α (**G**), and IL-18 (**H**) from cells was quantified by an ELISA assay. ^###^ *p* < 0.001 vs. LPS group. ^&&&^ *p* < 0.001 vs. LPS and LL-37 group.

Further analysis substantiated that LL-37 increased LPS-stimulated cell viability, which was reversed via ZBP1 overexpression (Figure 5A). Furthermore, the inhibitory effects of LL-37 on LDH release (Figure 5B), ROS production (Figure 5C), and MDA levels (Figure 5D) were offset after ZBP1 overexpression in the cells under LPS conditions. Concomitantly, LL-37-mediated anti-apoptotic efficacy in the LPS-treated cells was abrogated after ZBP1 elevation, as evidenced using an annexin V and TUNEL staining assay (Figure 5E,F). Moreover, LL-37 suppressed LPS-induced production of IL-1β, TNF-α (Figure 5G), and IL-18 (Figure 5H), which was reversed via ZBP1 overexpression.

### 2.6. Administration of LL-37 Alleviates Sepsis-Induced ALI with ZBP1 In Vivo

**Figure 6:**
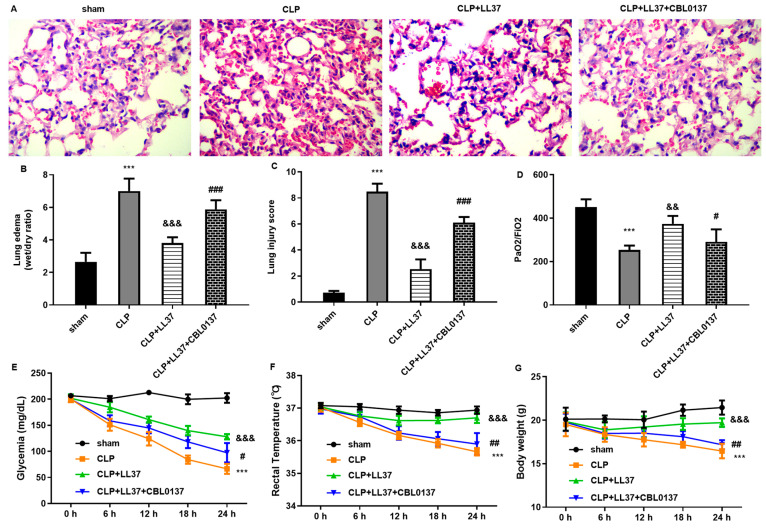
LL-37 alleviated lung injury in CLP mice by ZBP1. (**A**) Mice that underwent CLP were applied to construct a sepsis-induced ALI model. CLP mice were injected with the LL-37 or ZBP1 activator CBL0137. Then, HE staining was used to evaluate the pathological changes in lung tissues. (**B**–**D**) Lung edema (**B**), lung injury scores (**C**), and PaO2/FiO2 were then determined. (**E**–**G**) Then, glycemia contents (**E**), rectal temperature (**F**), and body weight (**G**) were detected in the mice under the various treatments. *** *p* < 0.001 vs. sham group. ^&&^ *p* < 0.05, ^&&&^ *p* < 0.001 vs. CLP group. ^#^ *p* < 0.05, ^##^ *p* < 0.01, ^###^ *p* < 0.001 vs. CLP and LL-37 group.

We next elucidated the function of LL-37 in sepsis-evoked ALI in vivo. Histopathological examination confirmed that the CLP-mimicking model showed obvious pathological alterations, including hemorrhage, inflammatory cell infiltration, interstitial edema, and diffuse alveolar damage, compared with the sham group (Figure 6A). Consistently, the CLP model had obvious lung tissue edema (Figure 6B) and high lung injury scores (Figure 6C). Intriguingly, LL-37 administration ameliorated CLP-induced histopathological changes, lung edema, and lung injury scores, which were reversed by ZBP1 activator CBL0137 (Figure 6A–C). Furthermore, LL-37 alleviated the CLP-induced reductions in PaO2/FiO2 (Figure 6D), glycemia contents (Figure 6E), rectal temperature (Figure 6F), and body weight (Figure 6G); however, these decreases were reversed after ZBP1 elevation.

### 2.7. LL-37 Ameliorates Inflammatory Response, Oxidative Injury, and ZBP1/Autophagy Signaling in Sepsis-Induced ALI Model

**Figure 7:**
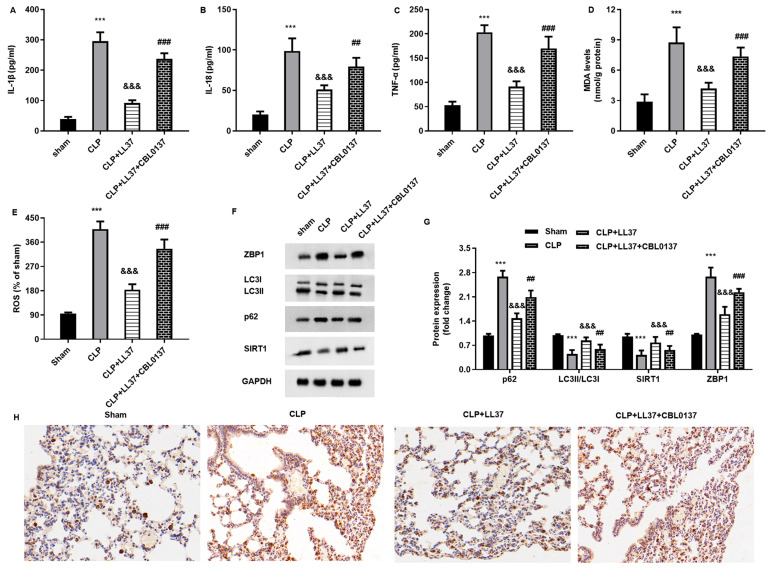
LL-37 restrained inflammation, oxidative injury, and ZBP1/autophagy signaling in the ALI model. (**A**–**C**) Mice that underwent CLP were injected with LL-37 and CBL0137. Then, the levels of IL-1β (**A**), IL-18 (**B**), and TNF-α (**C**) in serum were analyzed. (**D**,**E**) The contents of MDA (**D**) and ROS (**E**) were determined in lung tissues. (**F**,**G**) The protein expression of autophagy-related protein was evaluated. (**H**) The expression of ZBP1 in lung tissues was determined by an immunohistochemical assay. *** *p* < 0.001 vs. sham group. ^&&&^ *p* < 0.001 vs. CLP group. ^##^ *p* < 0.01, ^###^ *p* < 0.001 vs. CLP and LL-37 group.

LL-37 administration attenuated CLP-evoked elevation of IL-1β (Figure 7A), IL-18 (Figure 7B), and TNF-α (Figure 7C) levels in a serum of model mice. Moreover, the increased MDA (Figure 6D) and ROS levels (Figure 6E) were confirmed in lung tissues from the CLP mice, which were reversed by LL-37 treatment. Notably, the activation of ZBP1 with CBL0137 reversed the inhibitory efficacy of LL-37 in CLP-evoked oxidative stress (Figure 7D,E). Furthermore, the CLP-induced up-regulation of ZBP1 protein expression was abrogated by LL-37, which was further enhanced by CBL0137 (Figure 7F,G). Moreover, LL-37 enhanced autophagy-related protein LC3II and SIRT1 expression and inhibited p62 expression relative to the CLP groups, which was offset by CBL0137 (Figure 7F,G). Finally, an immunohistochemical assay revealed that the increased expression of ZBP1 in lung tissues from the CLP mice was suppressed by LL-37, which was enhanced by CBL0137 (Figure 7H).

## 3. Discussion

Sepsis-induced ALI usually leads to organ failure and constitutes a heavy burden on families and society due to high mortality and morbidity. A previous study revealed the therapeutic roles of LL-37 in septic mice [16]. Nevertheless, its roles in sepsis-evoked ALI are still unclear. LPS, a major component in Gram-negative bacteria, can induce systemic inflammation and microvascular lung injury. Therefore, LPS-induced injury has been recognized as a useful experimental model due to its similarities with the characteristics of ALI in humans [11,19,20]. In this research, LL-37 attenuated LPS-induced oxidative stress injury and inflammatory response in pulmonary epithelial cells. Moreover, LL-37 enhanced autophagy by inhibiting ZBP1 expression, which accounted for LL-37-mediated anti-oxidative injury and anti-inflammation in LPS-stimulated pulmonary epithelial cells. Importantly, in vivo, LL-37 alleviated CLP-induced ALI by blocking ZBP1 via ameliorating pathological changes, lung edema, and lung injury scores. Moreover, LL-37 inhibited oxidative stress and inflammation in the ALI mouse model, concomitant with autophagy activation. Thus, the current study highlights that LL-37 may represent a feasible therapeutic agent against sepsis-induced ALI by regulating ZBP-mediated autophagy activation.

Alveolar epithelial cells exert a critical function in antagonizing lung tissue injury and can establish a capillary alveolar epithelial barrier to prevent alveolar collapse and extra-vascular inflamed cell entrance into the alveolar space. It is generally believed that oxidative stress injury represents a known pathological mechanism in sepsis-induced ALI [12]. Under a sepsis-induced systemic inflammatory response, excessive inflammation leads to the overproduction of free radicals and induction of alveolar epithelial cell oxidative damage, which will further aggravate cell apoptosis and necrosis [12,20]. LPS, as a major component of Gram-negative bacteria, is a main cause of ALI and can be applied to mimic sepsis-induced ALI models in vitro [13,20]. Thus, we stimulated human alveolar pulmonary epithelial cells with LPS and confirmed oxidative stress injury after LPS stimulation. Interestingly, the LL-37 treatment alleviated LPS-evoked oxidative injury in alveolar pulmonary epithelial cells. Similarly, an emerging study also confirms that LL-37 attenuates heat-stroke-induced cytotoxicity in intestinal goblet cells by exerting its antioxidant function [21]. Several reports have revealed anti-oxidative stress as a potential approach for sepsis-induced ALI therapy [12,20]. In this study, LL-37 also attenuated oxidative stress levels in lung tissues from ALI mice. Thus, these findings reveal that LL-37 may attenuate the development of sepsis-induced ALI by suppressing oxidative injury.

Pathologically, the lung ranks as the most vulnerable organ after sepsis. Abundant inflammatory mediators caused by infection will induce alveolar epithelial cell injury and disrupt the alveolar microvascular barrier, which allows for inflammatory cell infiltration and evokes lung dysfunction manifested as ALI [10,12,20]. Currently, targeting the inflammatory response represents a promising therapeutic strategy for ALI caused by sepsis [9,20,22]. Therefore, before the investigation of LL-37 in ALI in vivo, we first constructed LPS-evoked inflammation in pulmonary epithelial cells. Consistent with a previous study [19,22], LPS stimulation evoked excessive inflammatory responses in alveolar pulmonary epithelial cells. Importantly, LL-37 restrained the above LPS-evoked inflammation. Moreover, in the ALI mouse model, LL-37 also suppressed CLP-increased inflammatory cytokines levels. Intriguingly, a previous study revealed that LL-37 could stimulate neutrophils to produce antimicrobial microvesicles to ameliorate pathological changes in sepsis [16]. Furthermore, LL-37 also inhibits contents of pro-inflammatory cytokines (TNF-α, IL-1β, and IL-6) in peritoneal fluids and sera in septic mice [23]. Importantly, the current findings substantiate that LL-37 alleviated CLP-evoked hemorrhage, inflammatory cells infiltration, diffuse alveolar damage, lung tissue edema, and injury score. Thus, LL-37 may attenuate the progression of ALI triggered by sepsis by inhibiting inflammation.

Accumulating evidence reveals that impaired autophagic–lysosomal degradation is responsible for lung tissue injury caused by sepsis [18,24]. Autophagy is an evolutionary conserved process that can exert a protective mechanism to degrade and recycle the damaged organelles. Under systemic inflammation after sepsis, autolysosomes are reduced in lung tissue and lead to autophagy dysfunction in ALI. Several studies confirm the dysregulation of autophagy in the lung tissues of patients with sepsis and in animal models [18,25]. Autophagy impairment can result in the accumulation of damaged mitochondria to induce ROS overproduction, which further aggravates oxidative injury and ALI progression [26]. Moreover, autophagy is implicated in pathophysiological process of sepsis-evoked ALI, including cell apoptosis and inflammatory response [27]. Intriguingly, LC3II-related autophagy was increased over 24 h in a CLP-induced ALI model; however, the impaired autophagosome–lysosome fusion gradually reduced within 24 h in sepsis-induced ALI [27]. Thus, increasing research supports that enhancing autophagy activation may attenuate the progression of ALI caused by sepsis [18,28]. LC3II is a standard marker for autophagosomes, and its high expression and down-regulation of autophagic substrate p62 indicate the activation of autophagy. In this study, a decreased expression of LC3II and increased expression of p62 were observed in LPS-treated pulmonary epithelial cells and lung tissues from the ALI model, indicating the impaired autophagy in the ALI model. Notably, LL-37 restored autophagy impairment in ALI cells and in the mice model, indicating that LL-37 may ameliorate the progression of ALI caused by sepsis by enhancing autophagy. Intriguingly, LL-37 can induce autophagy activation to eliminate live Porphyromonas gingivalis in keratinocytes [29]. Furthermore, RNA-seq sequencing analysis substantiated ZBP1 as a down-regulated gene with the largest fold change. Moreover, LL-37 treatment suppressed ZBP1 expression, whilst ZBP1 overexpression inhibited autophagy and reversed LL-37-mediated protection against LPS-induced oxidative injury and inflammation. Importantly, ZBP1 elevation reversed the protective function of LL-37 in lung injury in sepsis-caused ALI mice. Similarly, a previous study also revealed that ZBP2 might attenuate H_2_O_2_-induced cell autophagy in endothelial cells [30]. However, how does ZBP1 affect autophagy? It is unknown whether ZBP1 regulates autophagy through an autophagy-related signaling pathway (such as PI3K/AKT) or its domain. This is a limitation of our work and will be explored in the future.

## 4. Conclusions

Together, the current study highlights that LL-37 can ameliorate the progression of ALI caused by sepsis in in vitro and in vivo models by suppressing oxidative injury and the inflammatory response, concomitant with ZBP1 down-regulation and autophagy activation. Moreover, ZBP1-mediated elevation of autophagy may account for the protective efficacy of LL-37 against sepsis-induced ALI. Thus, these findings reveal a new mechanism regarding how LL-37 attenuates the progression of ALI caused by sepsis. Therefore, the current findings may support LL-37 as a promising therapeutic agent against ALI. However, the elevation of ZBP1 cannot completely block the protective efficacy of LL-37 against sepsis-induced ALI. How exactly does LL-37 reduce inflammation and oxidative stress injury in sepsis-induced ALI via ZBP1-mediated autophagy? Are there other pathways involved in the above process? Does LL-37 exert ideal efficacy in clinical practice? These questions will be addressed in our future work.

## 5. Materials and Methods

### 5.1. Cell Culture and Sepsis-Induced Lung Injury Model In Vitro

Human A549 pulmonary epithelial cells were bought from Shanghai Cell Collection (Shanghai, China). All cells were maintained in DMEM/F12 medium (Gibco, Waltham, MA, USA) containing 10% fetal bovine serum, 100 μg/mL streptomycin, and penicillin in a 5% CO_2_ incubator at 37 °C. To mimic sepsis-evoked lung injury, A549 cells were stimulated with lipopolysaccharide (LPS; 2 μg/mL; Sigma-Aldrich, St Louis, MO, USA) or LL-37 (50 μg/mL; Sangon Biotech, Shanghai, China) for 24 h in vitro.

### 5.2. Recombinant Plasmid Construction and Transfection

Before the construction of the ZBP recombinant vector, RNA was extracted from the A549 cells using commercial TRIzol Reagent (Invitrogen, Carlsbad, CA, USA). Then, first-strand cDNA was synthesized according to the protocols of a SuperScript™ IV First-Strand Synthesis System (Invitrogen). Subsequently, ZBP1 cDNA was obtained via PCR amplification and inserted into a pcDNA3.1(+) plasmid (Invitrogen) to prepare the ZBP1 recombinant vector pcDNA-ZBP1. After that, the cells were placed in six-well plates and transfected with empty or pcDNA-ZBP1 vector when cells had grown to 70–80% confluence. Forty-eight hours later, the efficacy of ZBP1 vector transfection was evaluated using Western blotting.

### 5.3. Cell Viability Detection

A549 cells, whether transfected with ZBP1 plasmids or not, were treated with LL-37 under LPS exposure (2 μg/mL) for 24 h. After that, all the specimens were collected and incubated with CCK-8 solution (10 μL; Nanjing Jiancheng Bioengineering Institute, Nanjing, China). Subsequently, the cell viability was measured at 4 h post-reaction when all specimens were analyzed using a spectrophotometer (Bio-Rad, Hercules, CA, USA) to measure the absorbance at 450 nm.

### 5.4. Detection of Lactate Dehydrogenase (LDH) Release

Following the treatment with plasmid or LL-37 under the LPS conditions, the A549 cell specimens were centrifuged for 15 min. Then, an LDH detection solution (Beyotime Biotechnology, Shanghai, China) was added into the prepared cell supernatant samples, shielding them from light for 30 min. The release of LDH was assessed by detecting OD490 nm.

### 5.5. Cell Apoptosis Analysis Using Flow Cytometer

After treatments under various conditions, the A549 cells were collected. Following rinsing with cold PBS (Sangon Biotech, Shanghai, China), 500 μL of binding buffer was added to re-suspend the collected samples (Beyotime, Shanghai, China). Then, annexin V-FITC (10 μL) and PI (5 μL) were added to the specimens at room temperature according to the instructions of a FITC-Annexin V Apoptosis Detection Kit. All specimens were then analyzed using a FACScan flow cytometer (BD Biosciences; San Jose, CA, USA).

### 5.6. TUNEL Staining

The treated A549 cells were fixed with 4% paraformaldehyde at room temperature. A total of 20 min later, the samples were rinsed with PBS three times, then re-suspended in PBS solution containing 0.3% of Triton X-100 (Sangon Biotech, Shanghai, China) and incubated for 5 min. Subsequently, all the specimens were reacted with TUNEL detection solution (50 μL) for 1 h at 37 °C in the dark. Finally, cells were analyzed under a fluorescence microscope (excitation wavelength of 450 nm and tracking emission wavelength of 520 nm).

### 5.7. qRT-PCR

After the synthesis of first-strand cDNA, real-time PCR was carried out on an ABI PRISM 7000 sequence detection system (Applied Biosystems, Foster City, CA, USA) to quantify the transcriptional expression of TNF-α, IL-18, IL-1β, p62, and SIRT1. All the protocols were constructed in reference to the instructions of a SYBR Premix Ex Taq^TM^ II Kit (Takara, Dalian, China). All primers were prepared by Sangon Biotech (Shanghai, China). The transcriptional levels of targeted genes were quantified via 2^−ΔΔCt^ and an internal control GAPDH.

### 5.8. Fluorescence Detection of Autophagy Level

To analyze the autophagy levels in A549 cells under various treatments, cell samples were infected with Ad-GFP-LC3B (Beyotime) at an MOI (Multiplicity of Infection) of 40. All the protocols were performed according to a commercial Ad-GFP-LC3B Kit, and all the samples were observed under a fluorescence microscope (Olympus, Tokyo, Japan).

### 5.9. RNA Transcriptome Sequencing Assay

A549 cells were stimulated with LPS and LL-37 for 24 h. The total RNA from the cells was extracted, purified using oligo dT magnetic beads and fragmented, and then applied to synthesize cDNA. Subsequently, a sequencing library was constructed using a DNA library construction kit (Illumina, San Diego, CA, USA), and the Illumina hiseq 2000 platform was applied for mRNA sequencing. After the data were taken off the plane, the FastQC software (version 0.11.8) was used for data quality control. Then, transcriptome data were compared using the HISAT2 software (version 2.2.1), and the gene expression was calculated using the Stringtie software (version 2.1.2) and Deseq2 (version 1.14.1). A heatmap and a volcano plot were used to analyze differentially expressed genes (DEGs) between both groups. DEGs are shown as a log2 fold change >1 and adjusted *p*-value < 0.05.

### 5.10. Experimental Animal and Ethics Statement

Male C57BL/6 mice (8 weeks, 20–25 g) were obtained from the Laboratory Animals Center, the Fourth Military Medical University. All mice were maintained in specific pathogen-free (SPF) conditions (12 h light/dark cycle at 22 ± 2 °C). All animals were fed with standard chow and autoclaved water for one week. All reasonable efforts were carried out to minimize suffering. All the experimental procedures were conducted following the National Institutes of Health (NIH) Guide for the Care and Use of Laboratory Animals, and all the experiments were approved via the Institutional Animal Care and Use Committee of West China Second University Hospital, Sichuan University.

### 5.11. Construction of Animal Model

Sepsis-associated ALI mice were constructed via CLP as previously described [19]. Mice were randomly divided into five groups (*n* = 5 in each group): the sham group, CLP group, CLP and LL37 group, and CLP + LL37 + CBL0137 (ZBP1 activator) group. For the CLP groups, mice were anesthetized intraperitoneally with pentobarbital sodium (60 mg/kg). Then, a 1 cm midline incision was generated in the lower abdomen to allow for cecum exposure. Subsequently, the cecum was ligated using 3-0 silk and then perforated with a 18-G needle to gently squeeze a droplet of feces into the peritoneal cavity to induce infection. The cecum was then repositioned and closed. After surgery, mice were resuscitated immediately through the subcutaneous injection of prewarmed normal saline (50 mL/kg). Mice in the sham groups underwent the same procedure without ligature and puncture. In the CLP and LL37 group, CLP model mice were intravenously injected with LL-37 (10 mg/kg). In the CLP + LL37 + CBL0137 (ZBP1 activator) group, CLP mice were intravenously injected with LL-37 (10 mg/kg) and CBL0137 (30 mg/kg). The weight, rectal temperature, and blood glucose were tested at 6 h intervals.

### 5.12. Detection of Oxygenation Index (PaO2/FiO2)

At 24 h after the operation, mice were anesthetized, and a 0.5 cm long incision was generated along the central axis of the anterior chest. Chest wall tissue was carefully separated to expose the chest cavity. Then, a 0.2 mL blood sample was collected from the heart using an arterial needle for blood oxygen analysis. Then, the oxygenation index (PaO2/FiO2) was calculated.

### 5.13. Hematoxylin and Eosin (HE) Staining and Lung Injury Score

Lung samples were collected and fixed with 4% paraformaldehyde. Then, all the specimens were embedded in paraffin. The tissue samples were cut into 5 μm thick serial sections. A histopathological evaluation was performed via HE staining, and lung injury scores were then assessed by two blinded pathologists, as previously reported [17].

### 5.14. Lung Edema Evaluation

To evaluate lung edema, the wet/dry weight (W/D) ratio was used. Briefly, the fresh upper portion of left lungs was excised and then rinsed with PBS. The weight of a wet lung was recorded. Then, the lung tissues were dried for 24 h at 80 °C in an oven, and the dry weight of the tissues was subsequently weighed.

### 5.15. Immunohistochemical Assay

For a histochemical analysis of ZBP1 expression in the lung tissues, lung sections were incubated with normal goat serum. One hour later, all samples were treated with anti-ZBP1 antibody (1:2000) at 4 °C overnight. Then, all section specimens were treated with biotin-labeled goat anti-rabbit secondary antibody at room temperature. Approximately 1 h later, slides were then developed with diaminobenzidine reagent (Zhongshan Company, Beijing, China) and counterstained with hematoxylin. The final images were observed using an Olympus BX53 microscope (Olympus, Tokyo, Japan).

### 5.16. Analysis of ROS and Malondialdehyde (MDA) Levels

To measure intracellular ROS levels in cells and tissue, an ROS Detection Kit (Nanjing Jiancheng Bioengineering Institute) was used. In brief, cells under various treatments were maintained in serum-free medium containing 10 µM 2′,7′-dichlorodihydrofluorescin diacetate (DCFH-DA). Approximately 0.5 h later, a microplate reader was used to measure the fluorescence intensity (with an excitation wavelength of 490 nm and tracking emission wavelength of 525 nm, respectively).

The contents of MDA in cells and lung tissues were determined using MDA Detection Kits (Nanjing Jiancheng Bioengineering Institute), following the manufacturer’s instructions. The absorbance at 450 nm was measured to quantify the levels of MDA.

### 5.17. Western Blotting Assay

The total protein from cells and tissue specimens was treated with RIPA lysis buffer. Subsequently, a BCA protein assay kit (Beyotime) was used to measure the prepared protein concentration. Then, approximately 30 μg of protein was subjected to 10% SDS-PAGE and transferred to a PVDF membrane. Following interdiction with non-fat dry milk (5%), the membrane was incubated with the primary antibody against LC3 (1:2000), p62 (1:20,000), SIRT1 (1:1000), and ZBP1 (1:1000) (all from Abcam, Cambridge, MA, USA) at 4 °C overnight. Subsequently, samples were hatched with horseradish-peroxidase-conjugated secondary antibodies. One hour later, binding bands were incubated with a chemiluminescent detection system and quantified using the Image J software (version 1.8.0).

### 5.18. Inflammatory Cytokine Detection via ELISA

The contents of inflammatory cytokines (TNF-α, IL-18, IL-1β) in the serum of mice and cell supernatant were detected using commercial ELISA kits (eBioscience, San Diego, CA, USA). All the procedures were conducted with reference to the manufacturer’s instructions.

### 5.19. Statistical Analysis

All experiments were carried out at least three times, and all data are shown as the mean ± standard deviation (SD). All statistical assays were analyzed using the SPSS19.0 software. A significant difference was determined using Student’s *t*-test for two groups and ANOVA with Tukey’s test for multiple groups. *p* < 0.05 was defined as statistically significant.

## Data Availability

The original contributions presented in this study are included in the article. Further inquiries can be directed to the corresponding author(s).

## References

[1] Hall M.J., Levant S., DeFrances C.J. (2013). Trends in inpatient hospital deaths: National Hospital Discharge Survey, 2000–2010. NCHS Data Brief..

[2] Bhojani N., Eisner B., Monga M., Paranjpe R., Cutone B., Chew B.H. (2023). Sepsis prevalence and associated hospital admission and mortality after ureteroscopy in employed adults. BJU Int..

[3] Lei S., Li X., Zhao H., Xie Y., Li J. (2022). Prevalence of sepsis among adults in China: A systematic review and meta-analysis. Front. Public. Health.

[4] Paoli C.J., Reynolds M.A., Sinha M., Gitlin M., Crouser E. (2018). Epidemiology and Costs of Sepsis in the United States—An Analysis Based on Timing of Diagnosis and Severity Level. Crit. Care Med..

[5] Sevransky J.E., Levy M.M., Marini J.J. (2004). Mechanical ventilation in sepsis-induced acute lung injury/acute respiratory distress syndrome: An evidence-based review. Crit. Care Med..

[6] Fan E., Brodie D., Slutsky A.S. (2018). Acute Respiratory Distress Syndrome: Advances in Diagnosis and Treatment. JAMA.

[7] Standiford T.J., Ward P.A. (2016). Therapeutic targeting of acute lung injury and acute respiratory distress syndrome. Transl. Res..

[8] Bos L.D.J., Ware L.B. (2022). Acute respiratory distress syndrome: Causes, pathophysiology, and phenotypes. Lancet.

[9] Kumar V. (2020). Pulmonary Innate Immune Response Determines the Outcome of Inflammation During Pneumonia and Sepsis-Associated Acute Lung Injury. Front. Immunol..

[10] Han S., Mallampalli R.K. (2015). The acute respiratory distress syndrome: From mechanism to translation. J. Immunol..

[11] Tang X., Liu J., Yao S., Zheng J., Gong X., Xiao B. (2022). Ferulic acid alleviates alveolar epithelial barrier dysfunction in sepsis-induced acute lung injury by activating the Nrf2/HO-1 pathway and inhibiting ferroptosis. Pharm. Biol..

[12] Bezerra F.S., Lanzetti M., Nesi R.T., Nagato A.C., Silva C.P.E., Kennedy-Feitosa E., Melo A.C., Cattani-Cavalieri I., Porto L.C., Valenca S.S. (2023). Oxidative Stress and Inflammation in Acute and Chronic Lung Injuries. Antioxidants.

[13] Chen R., Cao C., Liu H., Jiang W., Pan R., He H., Ding K., Meng Q. (2022). Macrophage Sprouty4 deficiency diminishes sepsis-induced acute lung injury in mice. Redox Biol..

[14] Durr U.H., Sudheendra U.S., Ramamoorthy A. (2006). LL-37, the only human member of the cathelicidin family of antimicrobial peptides. Biochim. Biophys. Acta.

[15] Suzuki K., Murakami T., Kuwahara-Arai K., Tamura H., Hiramatsu K., Nagaoka I. (2011). Human anti-microbial cathelicidin peptide LL-37 suppresses the LPS-induced apoptosis of endothelial cells. Int. Immunol..

[16] Nagaoka I., Tamura H., Reich J. (2020). Therapeutic Potential of Cathelicidin Peptide LL-37, an Antimicrobial Agent, in a Murine Sepsis Model. Int. J. Mol. Sci..

[17] Liu Q., Wu J., Zhang X., Li X., Wu X., Zhao Y., Ren J. (2021). Circulating mitochondrial DNA-triggered autophagy dysfunction via STING underlies sepsis-related acute lung injury. Cell Death Dis..

[18] Zhao H., Chen H., Xiaoyin M., Yang G., Hu Y., Xie K., Yu Y. (2019). Autophagy Activation Improves Lung Injury and Inflammation in Sepsis. Inflammation.

[19] Zheng Q., Wang Y.C., Liu Q.X., Dong X.J., Xie Z.X., Liu X.H., Gao W., Bai X.J., Li Z.F. (2020). FK866 attenuates sepsis-induced acute lung injury through c-jun-N-terminal kinase (JNK)-dependent autophagy. Life Sci..

[20] Zhou J., Peng Z., Wang J. (2021). Trelagliptin Alleviates Lipopolysaccharide (LPS)-Induced Inflammation and Oxidative Stress in Acute Lung Injury Mice. Inflammation.

[21] Shih C.C., Liao W.C., Ke H.Y., Kuo C.W., Tsao C.M., Tsai W.C., Chiu Y.L., Huang H.C., Wu C.C. (2023). Antimicrobial peptide cathelicidin LL-37 preserves intestinal barrier and organ function in rats with heat stroke. Biomed. Pharmacother..

[22] Xu B., Wang H., Chen Z. (2021). Puerarin Inhibits Ferroptosis and Inflammation of Lung Injury Caused by Sepsis in LPS Induced Lung Epithelial Cells. Front. Pediatr..

[23] Hu Z., Murakami T., Suzuki K., Tamura H., Reich J., Kuwahara-Arai K., Iba T., Nagaoka I. (2016). Antimicrobial cathelicidin peptide LL-37 inhibits the pyroptosis of macrophages and improves the survival of polybacterial septic mice. Int. Immunol..

[24] Qu M., Chen Z., Qiu Z., Nan K., Wang Y., Shi Y., Shao Y., Zhong Z., Zhu S., Guo K. (2022). Neutrophil extracellular traps-triggered impaired autophagic flux via METTL3 underlies sepsis-associated acute lung injury. Cell Death Discov..

[25] Yin X., Xin H., Mao S., Wu G., Guo L. (2019). The Role of Autophagy in Sepsis: Protection and Injury to Organs. Front. Physiol..

[26] Mohsin M., Tabassum G., Ahmad S., Ali S., Ali Syed M. (2021). The role of mitophagy in pulmonary sepsis. Mitochondrion.

[27] Zhao J., Liang Q., Fu C., Cong D., Wang L., Xu X. (2023). Autophagy in sepsis-induced acute lung injury: Friend or foe?. Cell Signal.

[28] Huang M., Yu Y., Tang X., Dong R., Li X., Li F., Jin Y., Gong S., Wang X., Zeng Z. (2023). 3-Hydroxybutyrate ameliorates sepsis-associated acute lung injury by promoting autophagy through the activation of GPR109alpha in macrophages. Biochem. Pharmacol..

[29] Yang X., Niu L., Pan Y., Feng X., Liu J., Guo Y., Pan C., Geng F., Tang X. (2020). LL-37-Induced Autophagy Contributed to the Elimination of Live *Porphyromonas gingivalis* Internalized in Keratinocytes. Front. Cell Infect. Microbiol..

[30] Jia Y., Chen X., Chen Y., Li H., Ma X., Xing W., Zhao K. (2021). Zhenbao pill attenuates hydrogen peroxide-induced apoptosis by inhibiting autophagy in human umbilical vein endothelial cells. J. Ethnopharmacol..

